# The New York Institute of Stomatology

**Published:** 1905-09

**Authors:** 

**Affiliations:** The New York Institute of Stomatology


### Reports of Society Meetings.

### THE NEW YORK INSTITUTE OF STOMATOLOGY.

A MEETING of the Institute was held at the Chelsea, No. 222 West Twenty-third Street, New York, on Tuesday evening, May 9, 1905, the president, Dr. C. O. Kimball, in the chair.

The minutes of the last meeting were read and approved. The subject for the evening was "Porcelain Inlays from the Clinical Stand-Point."

Dr. J. G. Palmer read a paper entitled "Porcelain Inlays from the Clinical Stand-Point—Cavity Preparation."

(For Dr. Palmer's paper, see page 582.)

Dr. F. L. Fossume read a paper on "The Matrix."

(For Dr. Fossume's paper, see page 588.)

Dr. L. A. O'Brian read a paper on "Building, baking, and finishing the Inlay."

(For Dr. O'Brian's paper, see page 584.)

DISCUSSION.

*Dr. H. W. Gillett.*—Dr. J. E. Nyman's recent article advocates the combination of high- and low-fusing porcelains. Dr. Nyman's device of a small depression in the floor of small cavities at one end where it would seem to define the position of the inlay, otherwise difficult to place correctly, is of value.

Dr. Ames requests that his cement be allowed to get wet as soon after the setting process begins. This being the case, why use varnishes or keep the patient sitting round with the rubber on.

I use silk or grass line for maintaining pressure on inlays, and think wedges likely to dislodge them from their exact seat. Dr. Head's suggestion of using a separator and then letting the tendency of the teeth to return to place hold the inlays in their proper position, is a valuable point to remember. I am not convinced as to the desirability of porcelain in the posterior teeth except for unusual conditions.

*Dr. H. L. Wheeler.*—It is especially interesting to hear one who was interested in making the formulæ of the Jenkins body so stoutly assert that the general principles pertaining to high- and low-fusing bodies are, as I have constantly maintained, that the higher the fusing point the better the color and the stronger the material.

The experiment of lining or making the first layers of the inlay of the "Close" body I have not tried, but I believe that this body covered with the Ash & Sons' high-fusing material would make an excellent and strong inlay with great possibilities in shade variations.

*Dr. E. A. Bogue.*—I showed Dr. Smith an inlay this afternoon that had been in twenty-five years. This inlay was set in the beginning with gum copal, but as that extruded the inlay, it was afterwards caulked on most of its circumference with gold. This porcelain was ground to fit its cavity. One method of holding small round inlays so as to find its proper position in the cavity is to attach it to the end of a stick with wax; it can then be returned to the cavity in the same position it occupied when the waxed stick picked it out.

I do not believe in inlays in crown cavities because they do not wear down evenly with the tooth, hence endanger the teeth later on when perhaps the patient cannot get relief.

I wish to call Dr. O'Brian's attention to the fact that feldspar will melt with all the appearance of glass, if heat enough be applied and the usual amount of potash be present. Hence it was not necessary to show the action of extreme heat on substances not designed to be subjected to such a degree of temperature.

The statement that porcelain fillings are "infinitely more lasting than gold operation hammered in" is also misleading. Time enough has not yet elapsed for us to know how lasting porcelain in approximal fillings may be. I have lately seen five gold fillings forty years old that were not hammered in, and three of those cases were pulpless teeth with the roots filled. These have lasted longer than any porcelain fillings, up to now.

*Dr. L. A. Faught.*—I am very much interested in this subject and have reached the conclusion that it is not necessary to undercut the inlays in setting. Etching them is sufficient.

One of my early experiences in the construction of porcelain tips was a case involving the six upper teeth. When I did this work I was somewhat of a pioneer, and I found difficulty in inducing the material to fuse so as to build up and out to the shape of the teeth. I think that the little forms which were exhibited here this evening, made by Ash & Sons, would have been a great help.

Adjourned.

FRED. L. BOGUE, M.D., D.D.S.,
*Editor The New York Institute of Stomatology.*

